# Supplementary figures and images for: The acute myeloid leukemia associated AML1-ETO fusion protein alters the transcriptome and cellular progression in a single-oncogene expressing in vitro induced pluripotent stem cell based granulocyte differentiation model
Source: PLoS One. 2019 Dec 23;14(12):e0226435. doi: 10.1371/journal.pone.0226435 (PMC6927605; doi:10.1371/journal.pone.0226435)

Figure S1

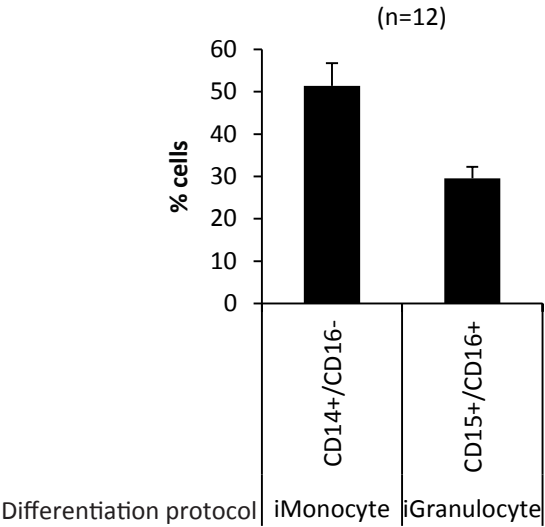

Supplement: S1 Fig — Flow cytometric analysis was performed on iPSCs differentiated towards the granulocyte or monocyte lineage (n = 12). Cells were stained with a cocktail of antibodies directed against CD45, CD14, CD16 and CD15. Cells were first gated on the CD45 leukocyte marker, followed by analysis of CD14-CD16 and CD15-CD16 to identify iMonocytes/iMacrophages and iGranulocytes, respectively. (PDF) [file pone.0226435.s001.pdf]

Figure S2

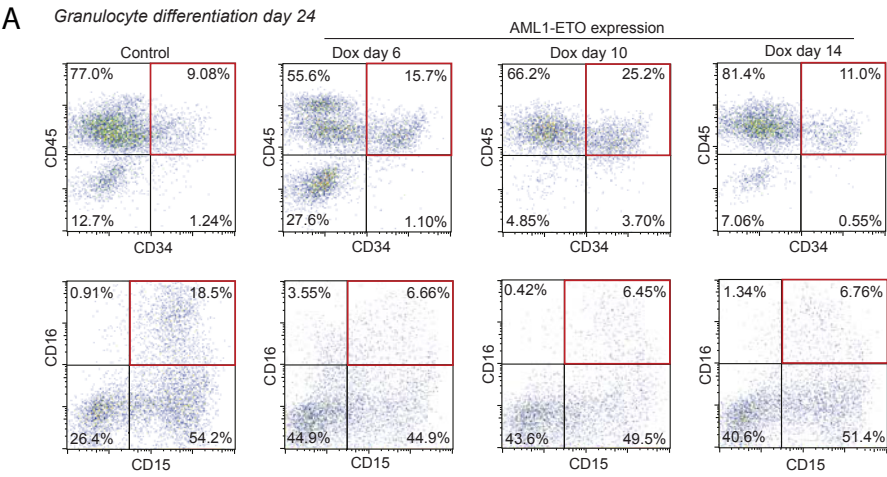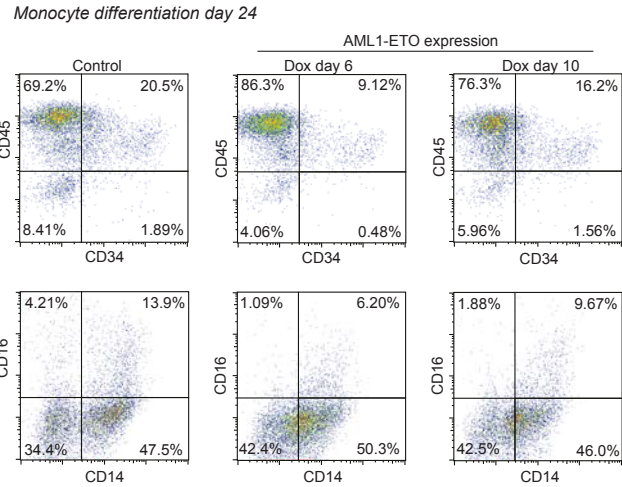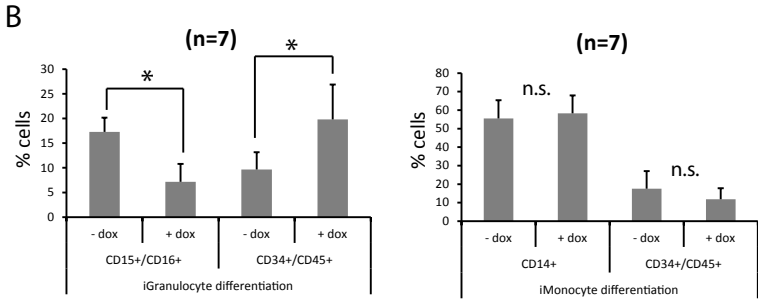

Supplement: S2 Fig — A. Flow cytometry analysis of control and AML1-ETO differentiated iPSCs towards the granulocytic and monocytic lineage. AML1-ETO was induced at day 0, 6, 10 and 14 of differentiation using doxycycline. Progenitor cells were characterized using CD34 and CD45 antibodies. To identify the iMonocytes/iMacrohages and iGranulocytes, cells were first gated on CD45 followed by analysis of CD14, CD16 and CD15 markers. B. Flow cytometry analysis of replicates (n = 7) of control and AML1-ETO differentiated iPSCs towards the monocytic (right) and granulocytic (left) lineage. Progenitor cells were characterized using CD34 and CD45 antibodies. To identify the iMonocytes/iMacrohages and iGranulocytes, cells were first gated on CD45 followed by analysis of CD14, CD16 and CD15 markers. * p-value <0.05, n.s. not significant. (PDF) [file pone.0226435.s002.pdf]

Figure S3

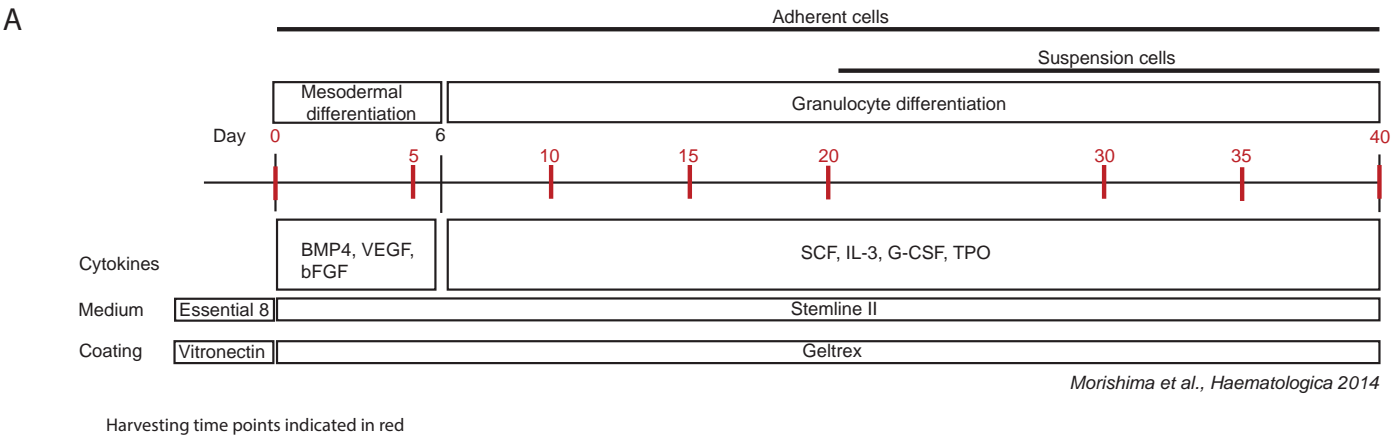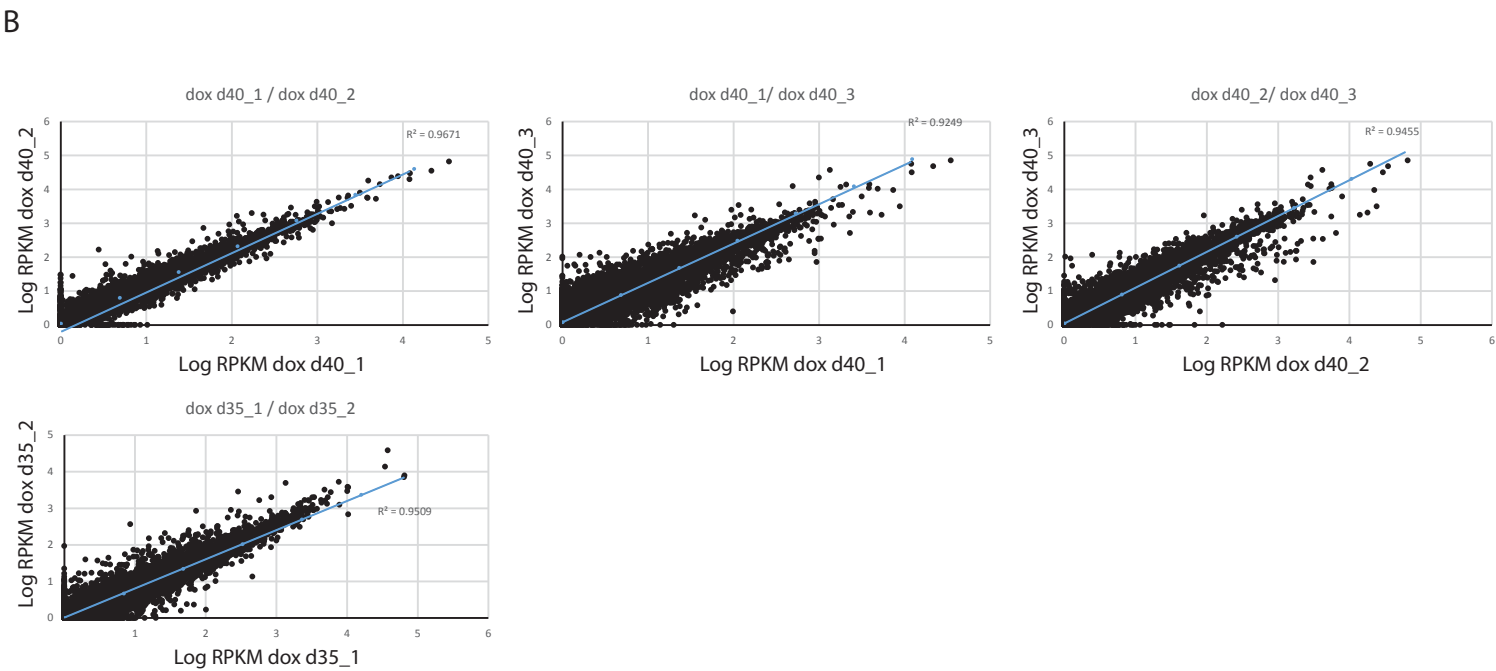

Supplement: S3 Fig — A. Schematic overview of hematopoietic differentiation towards the granulocytic lineage and the samples that were collected during hematopoietic differentiation of control and AML1-ETO induced cells. Adherent cells were collected from day 0–40 whereas suspension cells were collected from day 20–40. B. Scatterplot showing the log transformed normalized reads of replicate RNA-seq samples of the indicated time points. The blue line represents the trendline with the correlation coefficient depicted. (PDF) [file pone.0226435.s003.pdf]

Figure S4

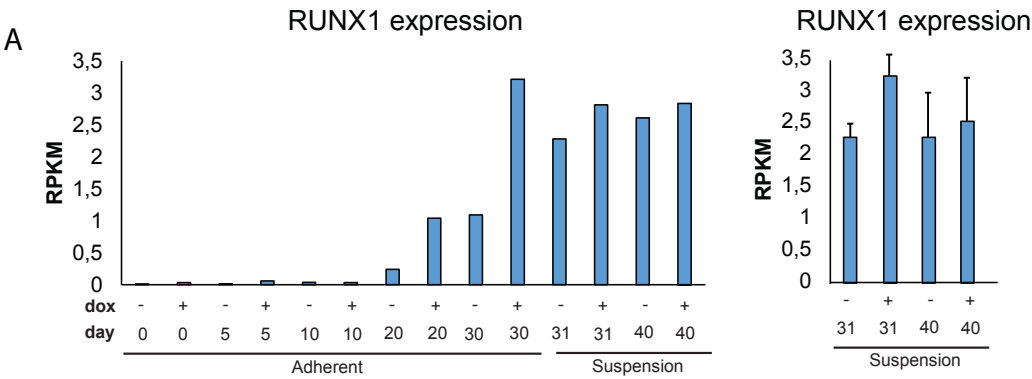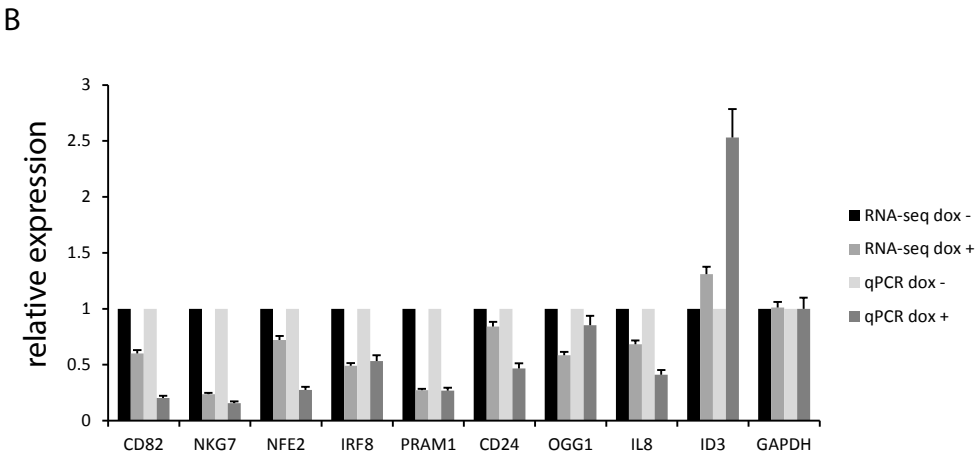

Supplement: S4 Fig — A. (Left) RPKM expression of RUNX1 gene in control (-dox) and AML1-ETO (+dox) differentiated iPSC at various time points during iGranulocyte development. (Right) Replicate analysis of RPKM expression of RUNX1 gene in control (-dox) and AML1-ETO (+dox) differentiated iPSC at two time points during iGranulocyte development. B. RT-qPCR validation of a subset of genes differentially expressed (based on the RNA-seq analysis) in AML1-ETO expressing and control differentiated iPSCs. For both the RT-qPCR experiment and the RNA-seq experiment the resulting values for the non-induced situation (–dox) for each gene was set at 1. The relative change according to this normalization is depicted in the figure. (PDF) [file pone.0226435.s004.pdf]

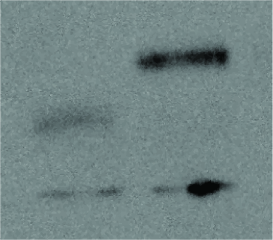

Supplement: S1 Raw Images — (TIF) [file pone.0226435.s008.tif]

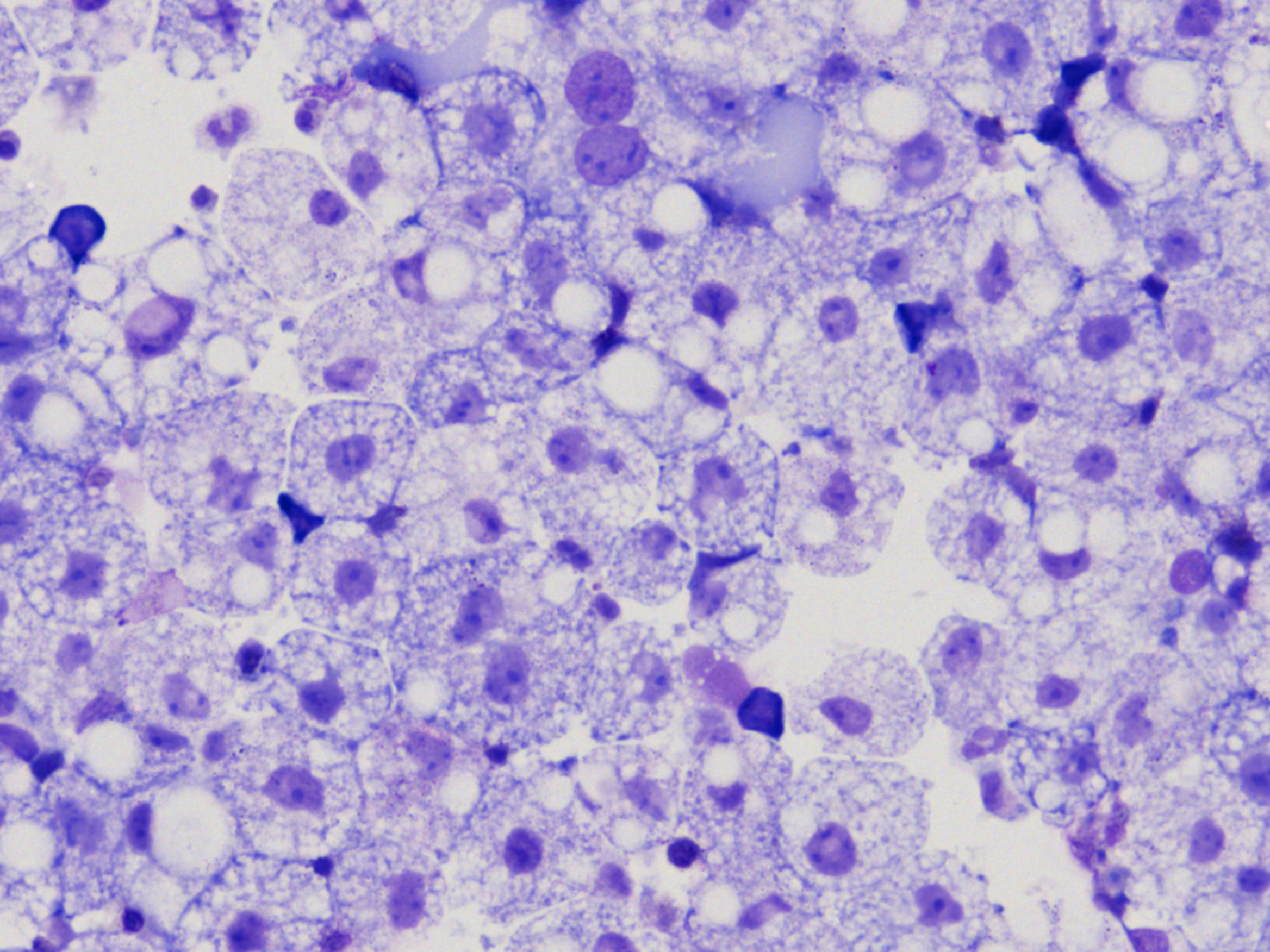

Supplement: S3 Raw Images — (TIF) [file pone.0226435.s010.tif]

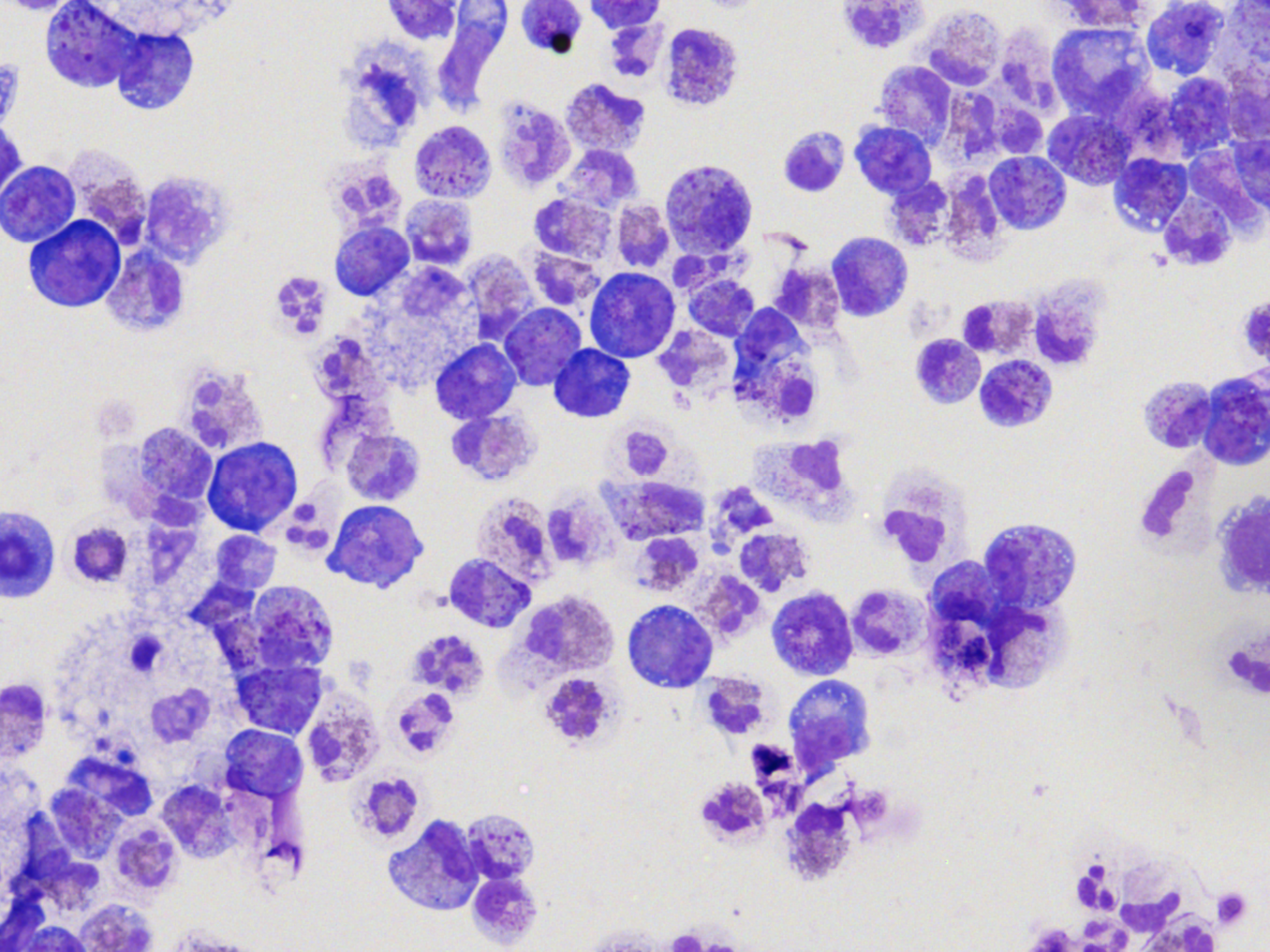

Supplement: S4 Raw Images — (TIF) [file pone.0226435.s011.tif]
