# Supplementary material for: The acute myeloid leukemia associated AML1-ETO fusion protein alters the transcriptome and cellular progression in a single-oncogene expressing in vitro induced pluripotent stem cell based granulocyte differentiation model
Source: PLoS One. 2019 Dec 23;14(12):e0226435. doi: 10.1371/journal.pone.0226435 (PMC6927605; doi:10.1371/journal.pone.0226435)
Supplement: S1 Table — (PDF) [file pone.0226435.s005.pdf]

**Supplemental Table 1**

*Gene Ontology Pathway Analysis*

Cluster 1

| Go term Biological Process | Binom Raw p-value |
|---|---|
| multicellular organism development | 1.48E-17 |
| developmental process | 1.96E-15 |
| regionalization | 5.90E-12 |
| cell differentiation | 8.97E-12 |
| cellular developmental process | 1.99E-11 |
| pattern specification process | 3.29E-11 |

Cluster 3

| Go term Biological Process | Binom Raw p-value |
|---|---|
| defense response | 2.38E-15 |
| inflammatory response | 1.11E-13 |
| response to stress | 1.15E-12 |
| immune response | 1.48E-12 |
| regulation of angiogenesis | 4.56E-09 |
| endocytosis | 3.08E-08 |

Cluster 4

| Go term Biological Process | Binom Raw p-value |
|---|---|
| system development | 3.01E-23 |
| multicellular organism development | 1.45E-22 |
| developmental process | 1.28E-20 |
| cell differentiation | 5.00E-18 |
| extracellular matrix organization | 2.82E-12 |
| cell fate commitment | 1.71E-08 |
