# Supplementary material for: The acute myeloid leukemia associated AML1-ETO fusion protein alters the transcriptome and cellular progression in a single-oncogene expressing in vitro induced pluripotent stem cell based granulocyte differentiation model
Source: PLoS One. 2019 Dec 23;14(12):e0226435. doi: 10.1371/journal.pone.0226435 (PMC6927605; doi:10.1371/journal.pone.0226435)
Supplement: S2 Table — (PDF) [file pone.0226435.s006.pdf]

Supplemental Table 2

Gene Ontology Pathway Analysis

Cluster 1

Go term Biological Process	Binom Raw p-value
immune system process	1.35E-26
cell activation	1.63E-25
signal transduction	8.10E-18
cell communication	1.20E-17
regulation of signaling	2.54E-12
regulation of cell differentiation	2.58E-12

Cluster 2

Go term Biological Process	Binom Raw p-value
cellular amino acid metabolic process	3.74E-10
cell cycle process	4.94E-09
organic acid metabolic process	1.49E-07
oxoacid metabolic process	1.67E-07
carboxylic acid metabolic process	9.13E-06
chromosome segregation	2.51E-05

Cluster 3

Go term Biological Process	Binom Raw p-value
immune response	1.15E-63
myeloid leukocyte activation	3.97E-49
granulocyte activation	7.02E-47
secretion by cell	2.74E-42
cell surface receptor signaling pathway	5.23E-24
inflammatory response	2.63E-21

Cluster 4

Go term Biological Process	Binom Raw p-value
wound healing	7.81E-13
hemostasis	2.50E-12
blood coagulation	4.18E-12
blood vessel development	1.31E-11
angiogenesis	9.47E-10
transport	2.52E-09

Cluster 5

Go term Biological Process	Binom Raw p-value
cell activation involved in immune response	2.88E-22
exocytosis	4.60E-18
vesicle-mediated transport	1.71E-17
pattern recognition receptor signaling pathway	2.44E-08
cell surface receptor signaling pathway	2.75E-08
regulation of phosphorus metabolic process	8.17E-08
