# Supplementary material for: The acute myeloid leukemia associated AML1-ETO fusion protein alters the transcriptome and cellular progression in a single-oncogene expressing in vitro induced pluripotent stem cell based granulocyte differentiation model
Source: PLoS One. 2019 Dec 23;14(12):e0226435. doi: 10.1371/journal.pone.0226435 (PMC6927605; doi:10.1371/journal.pone.0226435)
Supplement: S3 Table — (PDF) [file pone.0226435.s007.pdf]

Supplemental Table 3

Genes differentially expressed in AML1-ETO expressing iPSCs

A2M	CD82	FAM83A	IL1RN	MXD1	RNF149	STAB1
ABCG1	CDC42EP3	FCER1G	IL24	MYH10	RNU12	STAC
ABI3	CEACAM1	FCGBP	INS-IGF2	MYO1E	RNU4ATAC	STAR
ACP5	CECR6	FCGR3A	IPO11-LRRC70	NAIP	S100A12	STC2
ACSL1	CHAC1	FCGRT	IRF8	NCEH1	S100A6	SYNE2
ADAM19	CHDH	FCN1	ITGAM	NCF1	S100A9	TBC1D2
ADAP2	CHST15	FGR	ITGAX	NCF1B	SASH3	TBX20
ADGRE1	CKB	FLVCR2	ITGB7	NCF2	SCARNA16	TBX3
ADGRE2	CKLF	FMN1	KCNJ15	NEO1	SCARNA5	TDRD6
ADGRE3	CKLF-CMTM1	FMNL2	KCNK13	NLRC4	SCARNA6	TDRD9
ADGRG3	CLEC4A	FN1	KCNMA1	NOTCH1	SCUBE1	TFR2
ADM	CLEC5A	FOLR2	KCTD12	NRN1	SDC4	TG
AHSP	CLEC7A	FPR2	KLF1	NRP1	SEMA3F	TGFBI
ALDH1A2	COL1A1	FUCA1	KREMEN1	NRP2	SEMA6B	TIGAR
ALDH1L2	COL1A2	G0S2	KRT1	NSUN7	SERPINA1	TLR1
ALOX15	COL4A2	GAL3ST4	LACC1	NUAK1	SGK1	TLR2
ALOX5	COL5A1	GIMAP1-GIMAP5	LAT2	OAS3	SHANK1	TLR4
ALOX5AP	COL5A2	GIMAP5	LFNG	OLFML2B	SIGLEC5	TM4SF1
ALPL	COL6A1	GK	LGMN	OLFML3	SIGLEC7	TMEM26
AMICA1	COLEC12	GLT1D1	LILRA5	OLR1	SIGLEC8	TMIGD3
ANGPT1	CPA3	GNG12-AS1	LILRA6	OR8G1	SIGLEC9	TMTC1
ANK1	CPXM1	GNG2	LILRB2	OSBPL11	SIPA1L2	TNFSF11
ANKRD22	CR1	GPR34	LILRB3	OXER1	SKIL	TNFSF12
ANKRD34B	CREB5	GPR84	LILRB5	P2RY13	SLAMF8	TNFSF12-TNFSF13
ANOS1	CSF1	GPRIN3	LINC01094	P2RY6	SLC11A1	TNFSF13
AQP9	CSF2RB	GPT2	LOC100131635	PALD1	SLC15A3	TNS3
ASAH1	CSRP1	GRN	LOC100506585	PALM	SLC1A3	TREM2
ASGR2	CTSB	GTSF1	LOC101448202	PCOLCE2	SLC25A21	TRIB1
ASNS	CTSD	HAVCR2	LOC102724323	PDCD4	SLC26A11	TRPM2
ATF5	CTSH	HBE1	LPAR1	PFKFB2	SLC26A4	TRPM6
ATP8A2	CTSL	HBEGF	LPL	PFKFB3	SLC26A4-AS1	TRPS1
AZU1	CTSS	HBG1	LRP1	PHACTR3	SLC37A2	TSPAN15
B3GNT7	CUEDC1	HBG2	LRRC4	PHGDH	SLC43A1	TTYH3
BCL2A1	CXCL1	HIP1	LSP1	PKLR	SLC43A2	TUBA1A
BCL6	CYBB	HIST1H3I	LTB	PLA2G7	SLC6A6	UNC13A
BCL6B	CYFIP1	HK3	LY96	PLEC	SLC6A9	UPP1
BHLHE40	CYP1B1	HLA-B	MAF	PLEKHO1	SLC7A11	VGF
BHLHE41	CYSLTR2	HMOX1	MAFB	PLXDC2	SLC7A11-AS1	VLDLR-AS1
BIN1	CYTH4	HNMT	MAGED1	PLXNC1	SLC7A7	VNN2
BRI3	CYTL1	HOTS	MAP2K6	PMP22	SLC7A8	VSIG4
C1orf204	DAB2	HOXA10-HOXA9	MAPK13	PNPLA6	SLCO2B1	VWA5A
C1QA	DCN	HOXA9	MCEMP1	POSTN	SMAD7	WFDC1
C1QB	DCSTAMP	HOXB9	MERTK	PPARG	SMPDL3A	WLS
C1QC	DDIT4	HP	METTL9	PREX1	SNHG20	ZFPM2
C22orf34	DDIT4L	HPGDS	MGAM	PROCR	SNORA63	ZNF521
C3	DFNA5	HS3ST2	MGAT4A	PRSS33	SNORA74A	
C5AR1	DNAJC6	HSD17B14	MIR6516	PSAT1	SNORA8	
CASK	DOCK4	ID2	MME	PTCH2	SNORD15A	
CASP1	DOCK6	IER3	MMP12	QPCT	SOCS3	
CCDC125	DYSF	IER5L	MMP14	RAB20	SOWAHC	
CCR1	EGFL7	IFITM10	MMP9	RAB3IL1	SOX4	
CCR6	EPHB2	IFNGR1	MPEG1	RASSF4	SPARC	
CD1C	EREG	IGF2	MRC1	RBM47	SPRED1	
CD300LF	ETV5	IGFBP4	MS4A2	RBP7	SPTA1	
CD40	FAM20A	IGFBP5	MS4A6A	RETN	SPTBN5	
CD44	FAM20C	IGSF6	MS4A7	RGL1	ST14	
CD68	FAM65B	IL13RA1	MSR1	RIN2	ST6GALNAC1	
